# Involvement of the ERK/HIF-1α/EMT Pathway in XCL1-Induced Migration of MDA-MB-231 and SK-BR-3 Breast Cancer Cells

**DOI:** 10.3390/ijms22010089

**Published:** 2020-12-23

**Authors:** Ha Thi Thu Do, Jungsook Cho

**Affiliations:** College of Pharmacy and Integrated Research Institute for Drug Development, Dongguk University-Seoul, Dongguk-ro 32, Ilsandong-gu, Goyang, Gyeonggi 10326, Korea; doha201191@gmail.com

**Keywords:** chemokine, XCL1, XCR1, breast cancer cells, MDA-MB-231 cells, SK-BR-3 cells, cell migration, ERK, HIF-1α, EMT

## Abstract

Chemokine–receptor interactions play multiple roles in cancer progression. It was reported that the overexpression of X-C motif chemokine receptor 1 (XCR1), a specific receptor for chemokine X-C motif chemokine ligand 1 (XCL1), stimulates the migration of MDA-MB-231 triple-negative breast cancer cells. However, the exact mechanisms of this process remain to be elucidated. Our study found that XCL1 treatment markedly enhanced MDA-MB-231 cell migration. Additionally, XCL1 treatment enhanced epithelial–mesenchymal transition (EMT) of MDA-MB-231 cells via E-cadherin downregulation and upregulation of N-cadherin and vimentin as well as increases in β-catenin nucleus translocation. Furthermore, XCL1 enhanced the expression of hypoxia-inducible factor-1α (HIF-1α) and phosphorylation of extracellular signal-regulated kinase (ERK) 1/2. Notably, the effects of XCL1 on cell migration and intracellular signaling were negated by knockdown of XCR1 using siRNA, confirming XCR1-mediated actions. Treating MDA-MB-231 cells with U0126, a specific mitogen-activated protein kinase kinase (MEK) 1/2 inhibitor, blocked XCL1-induced HIF-1α accumulation and cell migration. The effect of XCL1 on cell migration was also evaluated in ER^-^/HER2^+^ SK-BR-3 cells. XCL1 also promoted cell migration, EMT induction, HIF-1α accumulation, and ERK phosphorylation in SK-BR-3 cells. While XCL1 did not exhibit any significant impact on the matrix metalloproteinase (MMP)-2 and -9 expressions in MDA-MB-231 cells, it increased the expression of these enzymes in SK-BR-3 cells. Collectively, our results demonstrate that activation of the ERK/HIF-1α/EMT pathway is involved in the XCL1-induced migration of both MDA-MB-231 and SK-BR-3 breast cancer cells. Based on our findings, the XCL1–XCR1 interaction and its associated signaling molecules may serve as specific targets for the prevention of breast cancer cell migration and metastasis.

## 1. Introduction

Despite advances in detection and treatment, breast cancer continues to be a worldwide healthcare burden. According to the Global Cancer Statistics (GLOBOCAN) 2018, breast cancer was the most commonly diagnosed cancer (24.2%) and the leading cause of cancer-related deaths (15.0%) in women [1]. Metastasis is considered a key contributor to breast cancer-associated mortality. According to Surveillance, Epidemiology, and End Results (SEER) 18 based on 2010–2016 data, the 5-year survival rate of breast cancer patients without metastasis exceeds 85% whereas the survival rate for metastatic cases is only 28.1%. Unfortunately, the effectiveness of current pharmacological therapies for breast cancer is generally limited. Thus, developing new strategies to effectively reduce breast cancer progression and metastasis is crucial [2]. Novel immunotherapies targeting the chemokine system, including chemokines and their receptors, are promising strategies for breast cancer therapy [2]. Therefore, current research has focused on elucidating the roles of the chemokine system in breast cancer metastasis.

Chemokines are a large family of chemotactic cytokines with low-molecular-weight (8 to 14 kDa) [3] and are classified into four subfamilies (CC, CXC, CX3C, and C chemokines) based on the relative positions of their N-terminal cysteine residues. Chemokines mediate their biological activities through interactions with their specific G protein-coupled receptors [4,5]. Chemokines and their receptors are well-known for their multiple roles in tumor progression, including immune responses, tumor growth and proliferation, angiogenesis, and metastasis [6]. X-C motif chemokine ligand 1 (XCL1; also known as lymphotactin), which is a member of the C chemokine subfamily, binds to its specific receptor X-C motif chemokine receptor 1 (XCR1) to mediate its effects [4,5]. The XCL1–XCR1 axis was reported to contribute to cell migration and proliferation of epithelial ovarian carcinoma (EOC) [7], non-small cell lung carcinoma (NSCLC) [8], and oral squamous cell carcinoma (OSCC) cells [9]. The role of XCR1 in breast cancer cells was also investigated using triple-negative MDA-MB-231 human breast cancer cells. In MDA-MB-231 cells, XCR1 overexpression inhibited tumor cell growth in vitro and tumorigenesis in vivo and promoted cell migration and invasion [10]. Moreover, although a reduced β-catenin level was suggested to be partly involved in the promotion of cell migration in XCR1-overexpressing cells, the exact underlying mechanisms of this phenomenon remain unclear. In this study, we studied the effect of XCL1 on breast cancer cell migration and further elucidated the intracellular signaling pathway mediating the biological activity of the XCL1–XCR1 axis.

Triple-negative breast cancers (TNBCs), which are characterized by a lack of estrogen receptor (ER), progesterone receptor, and human epidermal growth factor receptor 2 expression (HER2), account for 10–20% of breast cancer cases and are considered a poor prognostic factor due to their association with higher metastasis risk [11,12]. Furthermore, given that specific targets have not been identified, targeted therapies for TNBCs have not yet been established. A human TNBC cell line, MDA-MB-231, is commonly used as an in vitro model to investigate the effects and mechanisms of action of various agents on breast cancer cell migration, which is the first and a fundamental step for tumor metastasis [13,14,15]. Therefore, this cell line was used as a model in our study to evaluate the pro-migratory effect of XCL1. In addition to TNBCs, an ER^-^/HER2^+^ breast cancer subtype is generally associated with aggressive potency and high risks of metastasis and recurrence [16,17]. Hence, the effect of XCL1 on the migration of SK-BR-3 cells was also investigated and compared with its effect on MDA-MB-231 cell migration.

The chemokine system has been shown to promote cancer metastasis through various mechanisms, including hijacking cell migration pathways, the formation of a premetastatic niche, the establishment of pseudopodia, maintenance of cancer stem cell properties, and epithelial–mesenchymal transition (EMT) induction [6]. EMT is an important process in cancer metastasis in which epithelial cells lose either their cell polarity or cell–cell adhesion, and then gain migratory and invasive characteristics to become mesenchymal cells [18]. EMT is characterized by cellular and molecular changes, including downregulation of epithelial markers such as E-cadherin and upregulation of mesenchymal markers such as vimentin and N-cadherin [19]. Furthermore, activation of the Wnt/β-catenin pathway can trigger EMT, thereby causing cancer cell invasion [20]. Among many molecular signaling pathways associated with breast cancer cell migration, the nuclear translocation of β-catenin from the cytoplasm is responsible for the transmission of cellular signals into the nucleus, leading to the activation of numerous target genes that regulate EMT [21,22]. The EMT-induced enhancement of cancer cell migration and invasion is closely involved in tumor occurrence and infiltration as well as distant implantation [18]. Therefore, targeting EMT is thought to be a promising strategy for the development of anti-metastatic drugs against several types of cancer including breast cancer [23]. Interestingly, EMT can be modulated by hypoxia-inducible factor (HIF)-1α in either hypoxic or normoxic conditions [24,25]. The activation of Extracellular signal-regulated kinase (ERK) 1/2 can contribute to the induction of EMT via HIF-1α [26]. Numerous studies have linked increases in ERK1/2 phosphorylation with the promotion of EMT in metastatic breast cancer [27,28] as well as with enhanced migration and invasion of breast cancer cells [29,30,31]. Therefore, to elucidate the intracellular signaling pathway that mediates XCL1-induced breast cancer cell migration, the effects of the XCL1–XCR1 axis on EMT markers, ERK1/2 activation, and HIF-1α expression were also examined in MDA-MB-231 and SK-BR-3 cells in this study.

## 2. Results

### 2.1. Effect of XCL1 on MDA-MB-231 Cell Migration

**Figure 1:**
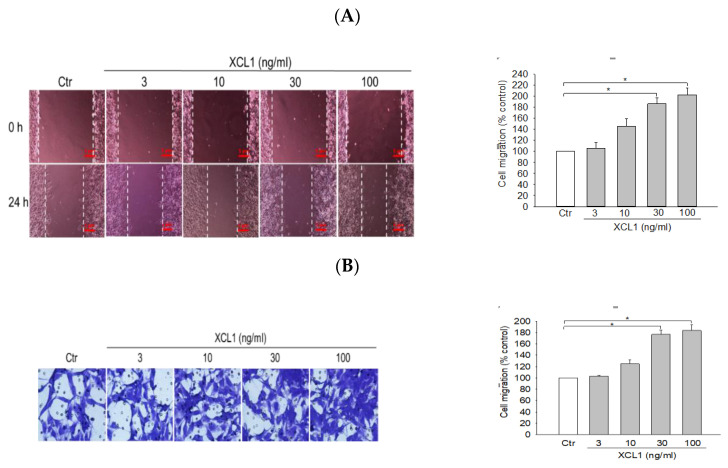
Pro-migratory effect of X-C motif chemokine ligand (XCL1) on MDA-MB-231 cells: MDA-MB-231 cells were treated with the indicated concentrations of XCL1 for 24 h. Control cells were treated with serum-free media instead. The differences in cell migration were analyzed as described in the Materials and Methods section using wound-healing (**A**) and transwell migration (**B**) assays. Representative images are shown. The data are displayed as the mean ± SEM from at least three independent experiments. * *p* < 0.05. Scale bar, 5 μm. Ctr, control.

To investigate the effect of XCL1 on MDA-MB-231 cell migration, the cells were treated with a series of XCL1 concentrations (3–100 ng/mL) for 24 h and cell migration was assessed via wound-healing and transwell migration assays. XCL1 treatment was found to markedly accentuate the migration of MDA-MB-231 cells in both assays (Figure 1). Particularly, an approximately 1.8–2-fold increase in cell migration was observed at XCL1 concentrations of 30 ng/mL and above. These results demonstrate that XCL1 has a pro-migratory effect on MDA-MB-231 cells.

### 2.2. Effect of XCR1 Knockdown on XCL1-Promoted MDA-MB-231 Cell Migration 

**Figure 2:**
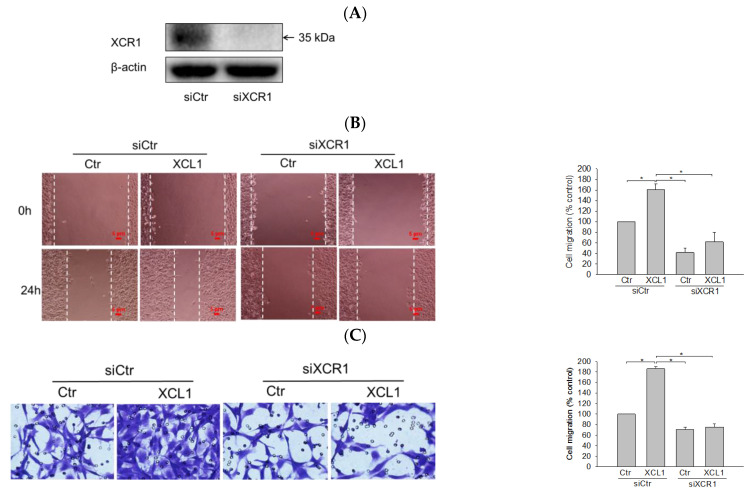
Abolition of XCL1-induced promotion of MDA-MB-231 cell migration by knockdown of X-C motif chemokine receptor 1 (XCR1): MDA-MB-231 cells were transfected with either siCtr or siXCR1, and Western blotting analysis was conducted using the anti-XCR1 antibody, as described in the Materials and Methods section. XCR1 expression was normalized to that of β-actin. A representative blot is shown (**A**). MDA-MB-231 cells were transfected with either siCtr or siXCR1 and treated with serum-free media (Ctr) or 100 ng/mL XCL1 for 24 h. The differences in cell migration were analyzed via the wound-healing (**B**) and transwell migration (**C**) assays. Representative images are provided. The data are displayed as the mean ± SEM from at least three independent experiments. * *p* < 0.05. Scale bar, 5 μm. Ctr, control.

XCL1 exerts its biological activity by binding to its specific receptor XCR1. To confirm that the XCL1-mediated accentuation of cell migration was through its interaction with XCR1, the cells were transfected with small interfering RNA (siRNA) targeting XCR1 (siXCR1) to knockdown the XCL1 receptor. Western blotting analyses showed complete inhibition of XCR1 expression in siXCR1-transfected cells compared to the cells transfected with control siRNA (siCtr) (Figure 2A). While XCL1 treatment prominently promoted the migration of siCtr-transfected MDA-MB-231 cells, the migration of cells transfected with siXCR1 was not significantly altered by XCL1 in both the wound-healing (Figure 2B) and transwell migration assays (Figure 2C). Therefore, our findings indicate that XCR1 is required for XCL1-induced promotion of MDA-MB-231 cell migration.

### 2.3. Effects of XCL1 on EMT in MDA-MB-231 Cells

#### 2.3.1. Effects of XCL1 on the Expression of EMT Markers in MDA-MB-231 Cells

**Figure 3:**
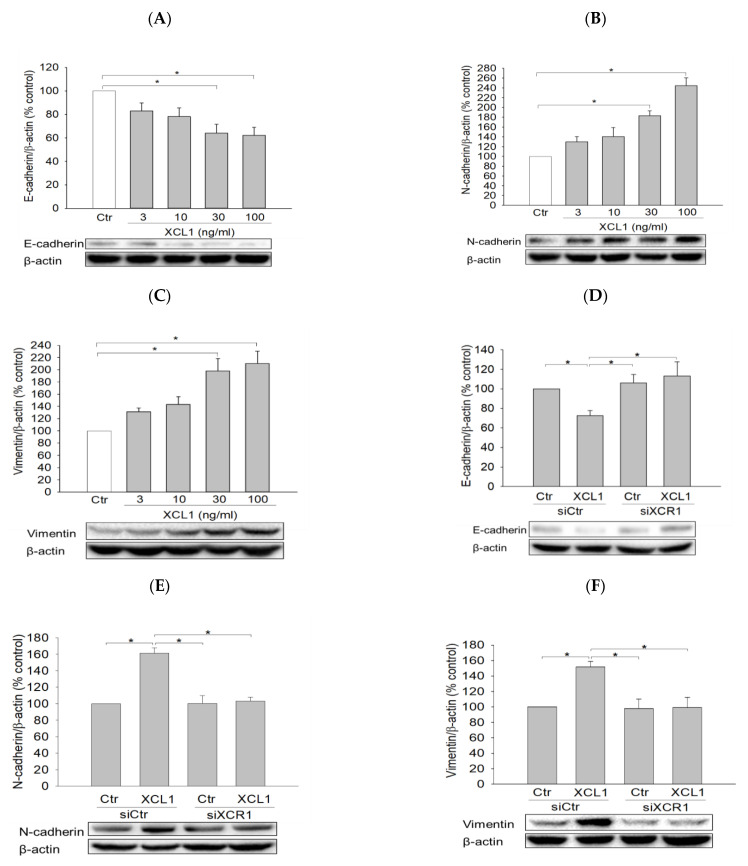
Effects of XCL1 on the expressions of E-cadherin and epithelial–mesenchymal transition (EMT) markers in MDA-MB-231 cells: MDA-MB-231 cells were treated with the indicated concentrations of XCL1 for 24 h (**A**–**C**). Control cells were treated with serum-free media instead. MDA-MB-231 cells were transfected with either siCtr or siXCR1 and treated with serum-free media (Ctr) or 100 ng/mL XCL1 for 24 h (**D**,**F**). Western blotting analyses were conducted using anti-E-cadherin (**A**,**D**), anti-N-cadherin (**B**,**E**), and anti-vimentin antibodies (**C**,**F**), as described in the Materials and Methods section. E-cadherin, N-cadherin, and vimentin expressions were normalized to that of β-actin. Representative blots are shown. The data are displayed as the mean ± SEM from at least three independent experiments. * *p* < 0.05. Ctr, control.

To elucidate the intracellular signaling pathway involved in XCL1-promoted MDA-MB-231 cell migration, we first investigated the effect of XCL1 on E-cadherin levels as well as various EMT markers including N-cadherin and vimentin. MDA-MB-231 cells were treated with a series of XCL1 concentrations for 24 h, after which protein expression levels were analyzed via Western blotting. We found that XCL1 concentration-dependently repressed E-cadherin expression (Figure 3A), whereas it dramatically increased the levels of N-cadherin and vimentin (Figure 3B,C). Similarly, XCL1 treatment reduced E-cadherin and enhanced N-cadherin and vimentin levels in siCtr-transfected MDA-MB-231 cells. In the MDA-MB-231 cells transfected with siXCR1, however, the expressions of E-cadherin, N-cadherin, and vimentin were not significantly altered by XCL1 treatment (Figure 3D–F). Our results demonstrate that EMT induced by XCL1 mediates MDA-MB-231 cell migration via interaction with XCR1.

#### 2.3.2. Effect of XCL1 on β-Catenin Nuclear Translocation in MDA-MB-231 Cells

**Figure 4:**
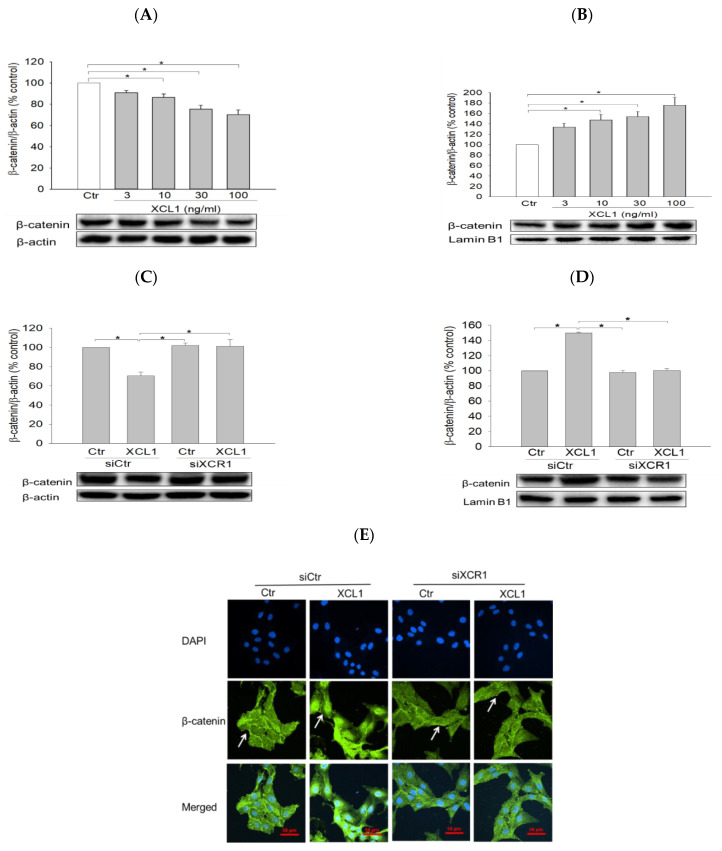
Enhancement of β-catenin nuclear translocation by XCL1 in MDA-MB-231 cells: MDA-MB-231 cells were treated with the indicated concentrations of XCL1 for 24 h (**A**,**B**). Control cells were treated with serum-free media instead. MDA-MB-231 cells were transfected with siCtr and siXCR1 and treated with serum-free media (Ctr) or 100 ng/mL XCL1 for 24 h (**C**,**D**). Western blotting analyses for cytosolic (**A**,**C**) and nuclear (**B**,**D**) extracts were conducted using anti-β-catenin antibody, as described in the Materials and Methods section. The expressions of cytosolic and nuclear β-catenin were normalized to those of β-actin and lamin B1, respectively. Representative blots are shown. The data are displayed as the mean ± SEM from at least three independent experiments. * *p* < 0.05. Representative immunofluorescence images from the immunocytochemical studies are provided (**E**). Scale bar, 50 μm. Ctr, control. The arrows indicate β-catenin subcellular localization.

We then examined the effect of XCL1 on the expression and subcellular localization of β-catenin in MDA-MB-231 cells. XCL1 treatment was found to decrease β-catenin levels in the cytoplasm and to increase it in the nucleus in concentration-dependent manners (Figure 4A,B). These results indicate that XCL1 enhances the nuclear translocation of β-catenin in MDA-MB-231 breast cancer cells, further evidencing that EMT is induced by XCL1. Consistent with the findings described above, XCL1 induced β-catenin nuclear translocation in MDA-MB-231 cells transfected with siCtr, which was completely abolished in the siXCR1-transfected MDA-MB-231 cells (Figure 4C,D). These findings were confirmed by immunocytochemical analysis (Figure 4E). Whereas β-catenin was strongly detected in the nuclei of XCL1-treated cells transfected with siCtr, it was only weakly detected in the nuclei of siXCR1-transfected cells treated with XCL1. The white arrows in Figure 4E indicate β-catenin subcellular localization. Our findings strongly suggest that the XCL1–XCR1 axis enhances cell migration by inducing EMT markers and β-catenin nuclear translocation in MDA-MB-231 breast cancer cells.

### 2.4. Effects of XCL1 on ERK/HIF-1α Signaling Pathway in MDA-MB-231 Cells

**Figure 5:**
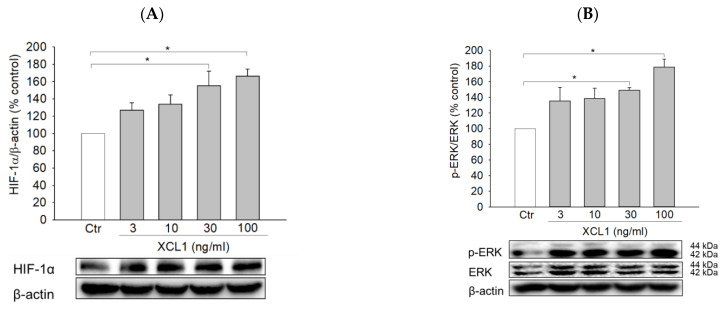

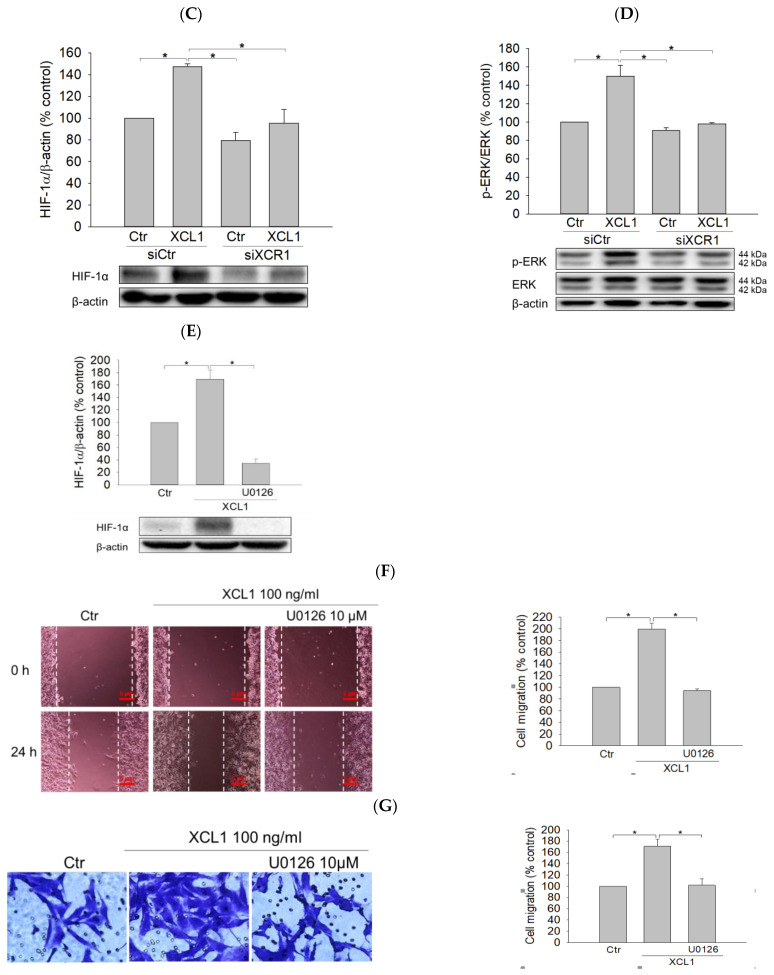
Activation of the Extracellular signal-regulated kinase (ERK)/hypoxia-inducible factor-1α (HIF-1α) signaling pathway by XCL1 in MDA-MB-231 cells: MDA-MB-231 cells were treated with the indicated concentrations of XCL1 for 24 h (**A**,**B**). Control cells were treated with serum-free media instead. MDA-MB-231 cells were transfected with either siCtr or siXCR1 and treated with serum-free media (Ctr) or 100 ng/mL XCL1 for 24 h (**C**,**D**). MDA-MB-231 cells were treated with serum-free media (Ctr) or 100 ng/mL XCL1 in the absence or presence of 10 μM U0126 for 24 h (**E–G**). Western blotting analyses were performed using anti-HIF-1α (**A**,**C**,**E**), anti-phospho-ERK1/2, and anti-ERK1/2 (**B**,**D**) antibodies, as described in the Materials and Methods section. The expressions of HIF-1α and ERK1/2 were normalized to that of β-actin. Representative blots are shown. The differences in cell migration were analyzed via the wound-healing (**F**) and transwell migration (**G**) assays. Representative images are provided. The data are displayed as the mean ± SEM from at least three independent experiments. * *p* < 0.05. Scale bar, 5 μm. Ctr, control.

As described above, our study determined that the induction of EMT was linked to the XCL1-promoted migration of MDA-MB-231 cells. To identify potential upstream signals regulating EMT induction, the effects of XCL1 on HIF-1α and ERK signaling were then studied. XCL1 was found to increase the expression of HIF-1α and phosphorylation of ERK1/2 (Figure 5A,B). These increases in HIF-1α and ERK1/2 phosphorylation were completely blocked by XCR1 knockdown in the siXCR1-transfected cells (Figure 5C,D). To examine whether activation of ERK1/2 was involved in the regulation of HIF-1α expression, the cells were treated with U0126, a specific inhibitor of mitogen-activated protein kinase kinase (MEK) 1/2, after which HIF-1α expression was evaluated. We found that U0126 (10 μM) markedly inhibited HIF-1α accumulation induced by XCL1 (Figure 5E), implying that ERK activation is the upstream signal that triggers HIF-1α accumulation to induce EMT in MDA-MB-231 cells. To confirm the involvement of ERK/HIF-1α signaling in the XCL1-induced promotion of MDA-MB-231 cell migration, we also tested the effect of U0126 on cell migration. As expected, U0126 treatment dramatically reduced XCL1-stimulated MDA-MB-231 cell migration (Figure 5F,G). These data indicate that the pro-migratory effect of XCL1 in MDA-MB-231 cells is mediated by activation of the ERK/HIF-1α signaling pathway. The ERK/HIF-1α signaling pathway is widely associated with EMT, which promotes invasion and metastatic dissemination of breast cancer cells [24,25,27,32,33]. In agreement with these reports, our results also demonstrate that the ERK/HIF-1α/EMT signaling pathway mediates XCL1-induced MDA-MB-231 cell migration.

### 2.5. Effects of XCL1 on the Expression of Matrix Metalloproteinases-2 and -9 in MDA-MB-231 Cells

**Figure 6:**
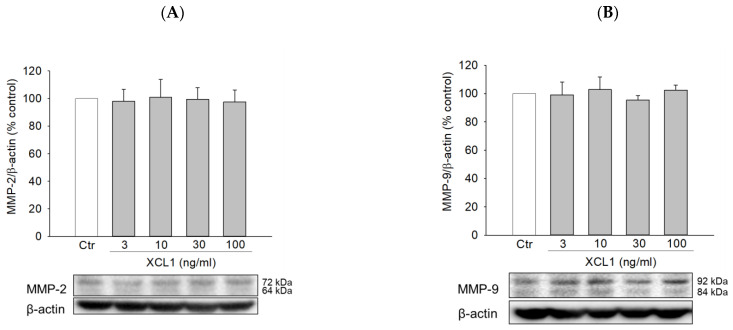
Effects of XCL1 on the expressions of matrix metalloproteinase (MMP)-2 and -9 in MDA-MB-231 cells: MDA-MB-231 cells were treated with the indicated concentrations of XCL1 for 24 h. Control cells were treated with serum-free media instead. Western blotting analyses were performed using anti-MMP-2 (**A**) and anti-MMP-9 antibodies (**B**), as described in the Materials and Methods section. The expressions of MMP-2 and -9 were normalized to that of β-actin. Representative blots are shown. The data are displayed as the mean ± SEM from at least three independent experiments. Ctr, control; MMP, matrix metalloproteinase.

Matrix metalloproteinases (MMPs), especially MMP-2 and -9, are known to participate in various phases of tumor progression, including cancer cell invasion and tumor metastasis [34,35,36]. Therefore, we evaluated if XCL1 had any impact on the expression of MMP-2 and -9 in MDA-MB-231 cells. We found that XCL1 did not cause any significant effects on MMP-2 and -9 at the concentrations tested in this study (Figure 6). These results suggest that MMP-2 and -9 signaling is not involved in the XCL1-induced promotion of MDA-MB-231 cell migration.

### 2.6. Effects of XCL1 on Cell Migration and the Intracellular Signaling Pathway in SK-BR-3 Breast Cancer Cells

**Figure 7:**
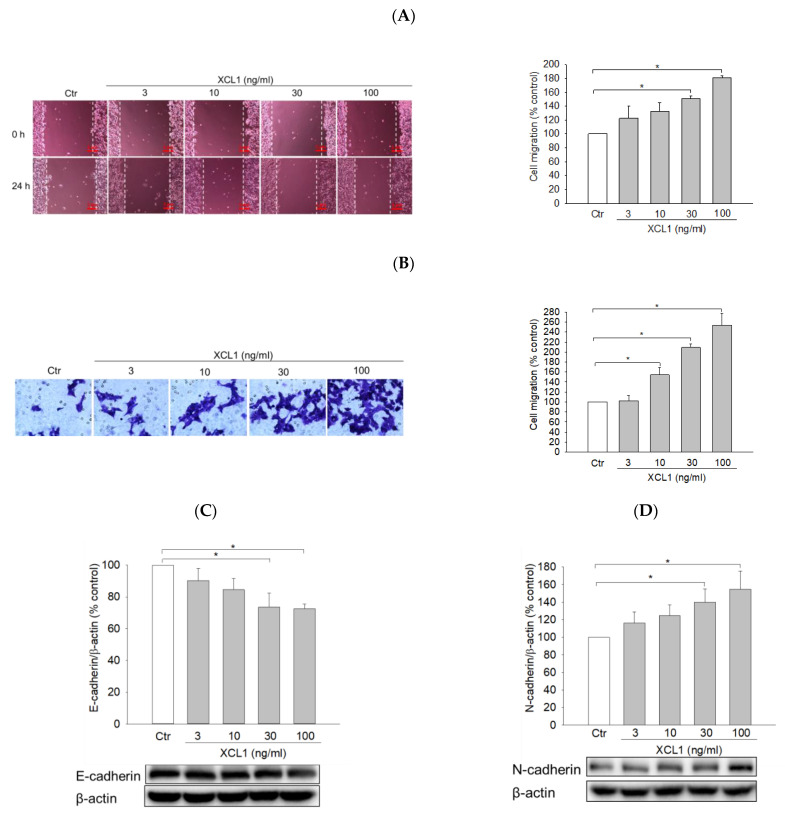

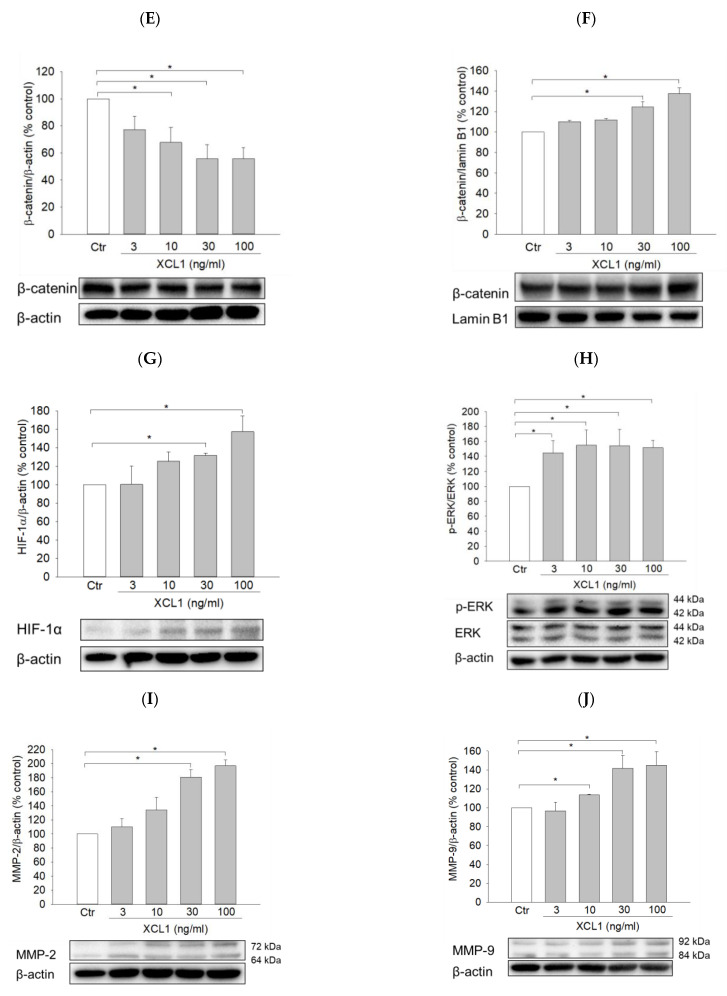
Effects of XCL1 on cell migration and intracellular signaling in SK-BR-3 cells: SK-BR-3 cells were treated with the indicated concentrations of XCL1 for 24 h. Control cells were treated with serum-free media instead. The differences in cell migration were analyzed as described in the Materials and Methods section using the wound-healing (**A**) and transwell migration (**B**) assays. Representative images are shown. Western blotting analyses were conducted using anti-E-cadherin (**C**), anti-N-cadherin (**D**), anti-HIF-1α (**G**), anti-phospho-ERK1/2 and anti-ERK1/2 (**H**), anti-MMP-2 (**I**), and anti-MMP-9 antibodies (**J**). Western blotting analyses of cytosolic (**E**) and nuclear (**F**) extracts were conducted using the anti-β-catenin antibody, as described in the Materials and Methods section. The expressions of E-cadherin, N-cadherin, cytosolic β-catenin, HIF-1α, ERK1/2, MMP-2, and MMP-9 were normalized to that of β-actin, whereas nuclear β-catenin expression was normalized to that of lamin B1. Representative blots are shown. The data are displayed as the mean ± SEM from at least three independent experiments. *p < 0.05. Scale bar, 5 μm. Ctr, control.

To explore whether the pro-migratory effect of XCL1 is specific for MDA-MB-231 cells or also applicable to the other type of breast cancer cells, we investigated the effects of XCL1 on cell migration and intracellular signaling in SK-BR-3 cells. XCL1 concentration-dependently enhanced SK-BR-3 cell migration in both wound-healing and transwell migration assays (Figure 7A,B). Although XCL1 did not change the expression of vimentin (data not shown), it significantly affected other EMT markers. XCL1 reduced E-cadherin expression and increased N-cadherin and β-catenin nuclear translocation (Figure 7C–F), indicating EMT induction by XCL1 in SK-BR-3 cells. Moreover, XCL1 accentuated HIF-1α accumulation and ERK1/2 phosphorylation in this breast cancer cells (Figure 7G,H). These results indicate that activation of the ERK/HIF-1α/EMT pathway is also involved in XCL1-induced SK-BR-3 cell migration.

Interestingly, however, while XCL1 had no significant effect on MMP-2 and -9 expression in MDA-MB-231 cells (Figure 6), this chemokine increased the expression of these proteinases in SK-BR-3 cells (Figure 7I,J). Therefore, in addition to activation of the ERK/HIF-1α/EMT pathway, the increased levels of MMP-2 and -9 expression may also mediate XCL1-induced SK-BR-3 cell migration.

### 2.7. Effects of XCL1 on the ERK/HIF-1α/EMT Signaling Pathway in A549 NSCLC and Panc1 Pancreatic Cells

**Figure 8:**
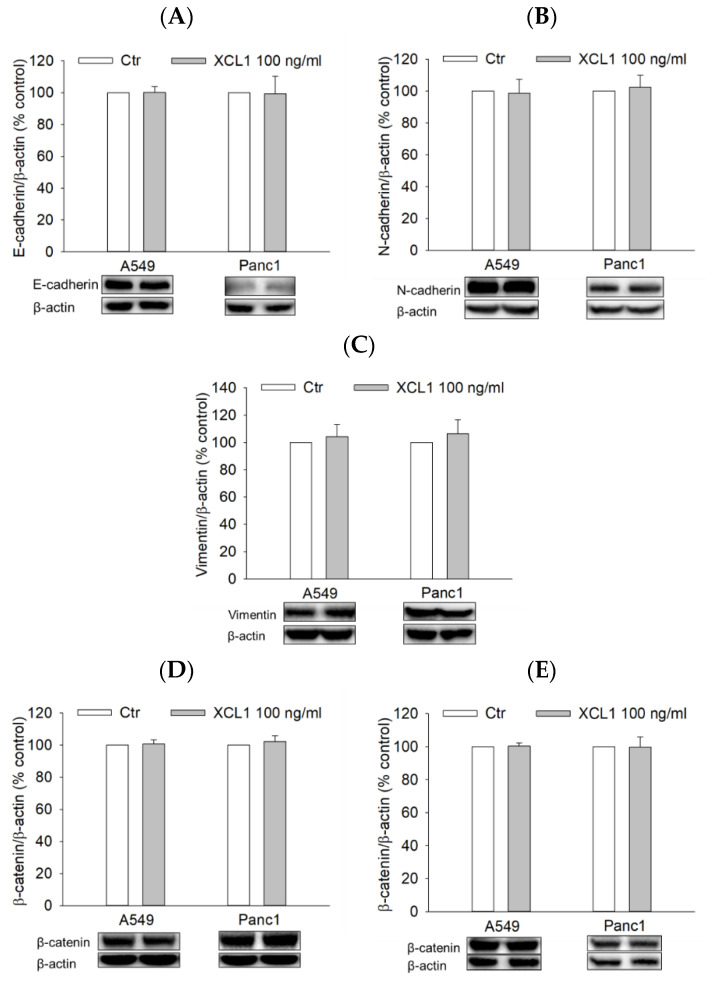

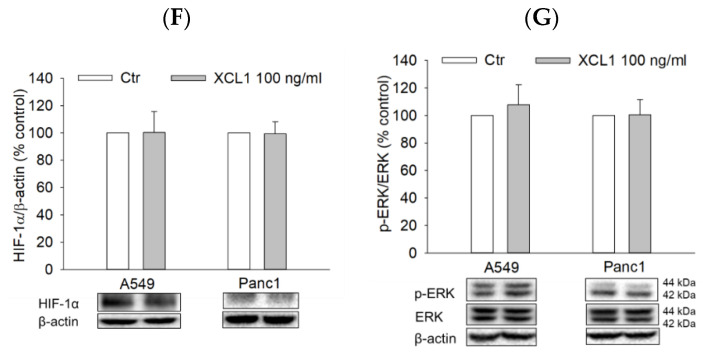
Effects of XCL1 on ERK/HIF-1α/EMT in A549 and Panc1 cells: A549 and Panc1 cells were treated with 100 ng/mL XCL1 for 24 h. Control cells were treated with serum-free media instead. Western blotting analyses were conducted using anti-E-cadherin (**A**), anti-N-cadherin (**B**), anti-vimentin (**C**), anti-HIF-1α (**F**), anti-phospho-ERK1/2, and anti-ERK1/2 antibodies (**G**). Western blotting analyses of cytosolic (**D**) and nuclear (**E**) extracts were conducted using anti-β-catenin antibody, as described in the Materials and Methods section. The expressions of E-cadherin, N-cadherin, vimentin, cytosolic β-catenin, HIF-1α, and ERK1/2 were normalized to that of β-actin, whereas nuclear β-catenin expression was normalized to that of lamin B1. Representative blots are shown. The data are displayed as the mean ± SEM from at least three independent experiments. Ctr, control.

To examine whether XCL1 also activated the ERK/HIF-1α/EMT signaling pathway of other cancer cell types, A549 NSCLC and Panc1 pancreatic cancer cells were treated with 100 ng/mL XCL1 for 24 h and assessed for EMT markers as well as HIF-1α and ERK1/2. In both A549 and Panc1 cells, however, XCL1 did not show any effect on E-cadherin or EMT markers, including N-cadherin, vimentin, and β-catenin (Figure 8A–E). Similarly, XCL1 had no impacts on HIF-1α and ERK phosphorylation (Figure 8F,G). These results suggest that XCL1 has no significant effects on these signals in A549 and Panc1 cells at the concentration tested in this study.

## 3. Discussion

Chemokines and chemokine receptors play crucial roles in the progression of breast cancer, including its occurrence, immune responses, angiogenesis, metastasis, and drug resistance [2]. Our study aimed to evaluate the roles of XCL1 and its specific receptor XCR1 in MDA-MB-231 cell migration as well as to identify the underlying signaling pathway mediating this process. Our data indicated that XCL1 accentuated the migration of MDA-MB-231 cells through its interaction with XCR1. Notably, the pro-migratory effect of the XCL1–XCR1 interaction was mediated by activation of the ERK/HIF-1α/EMT signaling pathway.

We found that XCL1 promoted MDA-MB-231 breast cancer cell migration in a concentration-dependent manner (Figure 1). The XCL1-induced promotion of MDA-MB-231 cell migration was inhibited by the knockdown of XCR1 (Figure 2), confirming that XCL1 activity is dependent on XCR1. It has been previously reported that XCR1 mRNA expression in MDA-MB-231 cells is lower than that in the other breast cancer cell lines such as MCF-7 cells [10]. Based on our findings, however, MDA-MB-231 cells may express sufficient levels of XCR1 to mediate the effects of XCL1. Despite the low level of XCR1 mRNA expression, XCR1 overexpression promoted MDA-MB-231 cell migration and invasion [10]. Similar results showing XCR1-mediated cell migration were reported in other cancer cells including EOC [7], NSCLC [8], and OSCC [9]. However, the mechanisms mediating cancer cell migration varied depending on cell type. For example, these effects may involve the Janus tyrosine kinase 2 (JAK2)/signal transducer and activator of transcription 3 (STAT3) cascade including MMP-2 and -9 in NSCLC cells [8] and the activation of ERK signaling in OSCC cells [9].

MDA-MB-231 cells, a human TNBC cell line, possess characteristics of mesenchymal cells with highly migratory and invasive capacities [37,38]. The increased migration of XCR1-overexpressing MDA-MB-231 cells may be through the alleviation of β-catenin [10]. This finding led us to hypothesize that the effect of the XCL1–XCR1 axis on MDA-MB-231 cell migration may be associated with EMT enhancement. Therefore, we examined the impacts of this chemokine axis on the expression of EMT markers in MDA-MB-231 cells.

EMT is a convertible and fundamental process that governs morphogenesis in multicellular organisms. This process is associated with a dedifferentiation program that results in dissemination of single carcinoma cells from the original location of the primary tumors to distant sites, which leads to malignancy [39]. During the EMT process, cancer cells lose their epithelial cell characteristics, such as polarity and cell adhesion, trigger remodeling of their actin cytoskeleton, acquire a mesenchymal cell-like morphology, and eventually accentuate their migratory ability [39,40]. Once cancer cells arrest the expression of epithelial markers including E-cadherin, the expression of mesenchymal markers such as N-cadherin and vimentin becomes upregulated [40]. The shift from E-cadherin to N-cadherin expression is crucial in EMT and cellular motility processes [41] and is correlated with the increased migratory and invasive properties of breast cancer cells [42,43]. Vimentin is a member of the intermediate filament family, which constitutes a part of the cytoskeleton. Increased vimentin expression is an indicator of highly migratory cells [44]. Moreover, E-cadherin plays an essential role in β-catenin function and stabilization via formation of the E-cadherin/β-catenin complex in the cytoplasm. This complex is critical for cell–cell adhesion and maintenance of the epithelial phenotype. Upon E-cadherin suppression, β-catenin can be disassociated from the E-cadherin/β-catenin complexes, can translocate to the nucleus, and can then transcriptionally activate various pro-migratory genes for EMT induction [45]. Therefore, loss of E-cadherin may increase the nuclear localization of β-catenin and enhance EMT in breast cancer cells [19,42]. Interestingly, EMT is a central mechanism to generate tumor-initiating cells (TICs, also known as cancer stem cells), which drive tumor initiation, progression, and metastasis. Especially, a whole transcriptome sequencing data of migratory versus non-migratory MDA-MB-231 cells revealed a significant enrichment of TICs in the migratory cell population and confirmed the fundamental link between EMT and TICs in breast cancer migration [46]. Our study determined that XCL1 downregulated E-cadherin expression, upregulated N-cadherin and vimentin, and promoted β-catenin nuclear translocation in MDA-MB-231 cells in concentration-dependent manners. All effects on these EMT markers were inhibited by XCR1 knockdown via siXCR1 transfection (Figure 3 and Figure 4). Our results confirmed and complemented previous findings [10], thereby demonstrating the involvement of EMT in XCL1-promoted MDA-MB-231 cell migration.

We investigated further to identify the signaling pathway associated with the effects of the XCL1–XCR1 axis on cell migration and EMT promotion. XCL1 was found to enhance HIF-1α accumulation in a concentration-dependent manner, and XCR1 knockdown with siXCR1 blocked this effect in MDA-MB-231 cells (Figure 5A,C). HIF-1α is a transcriptional factor for many genes that code for various proteins involving tumor cell proliferation and survival, angiogenesis, glucose metabolism, and invasion and metastasis pathways [19,47]. HIF-1α is a known regulator of metastatic niche formation in breast cancer [48] and the development of lung metastasis in TNBC [49]. Increasing evidence has indicated that high levels of HIF-1α are likely indicative of poor clinical prognosis in breast cancer patients with lymph node-negative disease [50] and invasive breast carcinoma [51]. HIF-1α can be regulated by either oxygen-dependent or -independent mechanisms. Particularly, the expression of HIF-1α is enhanced under hypoxic conditions in all cell types, which is mainly associated with suppressed HIF-1α degradation [47]. On the other hand, growth factors, cytokines, and other signaling molecules can mediate HIF-1α synthesis in a cell-type-specific manner through the activation of phosphatidylinositol 3-kinase (PI3K) or mitogen-activated protein kinase (MAPK) pathways [47,52,53,54]. Furthermore, the link between HIF-1α accumulation and EMT/cancer cell migration has been well established in both hypoxic and normoxic conditions. EMT can be modulated by HIF-1α via the regulation of EMT transcription factors such as TWIST, SNAIL, SIP1, SLUG, and ZEB1 or cross-talk with the transforming growth factor-β/SMAD, Wnt/β-catenin, Notch, and nuclear factor kappa B pathways [24,25]. For example, hypoxia or overexpression of HIF-1α promotes EMT through the regulation of TWIST expression by directly binding to the hypoxia-response element in the TWIST proximal promoter, which leads to tumor progression and metastasis [52]. The distinct transcriptome profile of MDA-MB-231 cells revealed very similar gene expression data between migratory cells. Particularly, pathways related to EMT, including the HIF-1α transcription factor network, were strongly associated with migratory breast cancer cells [55]. In our study, XCL1 not only enhanced cell migration and promoted EMT in MDA-MB-231 cells but also enhanced HIF-1α accumulation. These results strongly suggest that the accumulation of HIF-1α mediates XCL1-induced EMT in MDA-MB-231 cells. It has been previously reported that the activation of ERK signaling is involved in triggering HIF-1α-induced EMT in lung cancer cells [26]. Therefore, we next investigated whether XCL1 had any effect on the activation of ERK signaling in MDA-MB-231 cells.

XCL1 was found to increase the phosphorylation of ERK1/2 in MDA-MB-231 cells, which was inhibited in siXCR1-transfected cells (Figure 5B,D). The effect of XCL1 on ERK activation in MDA-MB-231 cells was similar to that in H357 and SCC4 oral cancer cells. Exposure of these cells to XCL1 significantly enhanced intracellular ERK1/2 phosphorylation and stimulated cell proliferation, migration, and invasion [9]. Interestingly, although XCR1 overexpression accentuated MDA-MB-231 cell migration, it was reported to suppress cell proliferation, tumorigenesis, and tumor growth, probably via the inhibition of MAPK activation with the reduced phosphorylation of MEK, JNK, and p38-MAPK [10]. In our study, treating MDA-MB-231 cells with XCL1 at a series of XCL1 concentrations (3–100 ng/mL) for 24 h did not cause any significant effect on cell proliferation (data not shown). The discrepancy between our findings and the previous report [10] on ERK1/2 phosphorylation and MDA-MB-231 cell proliferation may be due to inherent differences in the experimental models. Specifically, Yang et al. examined the effect of XCR1 overexpression in MDA-MB-231 cells [10], whereas we assessed the effect of XCL1 treatment using naïve MDA-MB-231 cells. Further investigation may be necessary to elucidate the possible reason(s) for these discrepancies. Nonetheless, we confirmed the involvement of XCL1-induced ERK activation in HIF-1α accumulation as well as the promotion of cell migration in naïve MDA-MB-231 cells using U0126, a specific MEK1/2 inhibitor.

Our study determined that the inhibition of ERK1/2 activation by U0126 completely blocked XCL1-induced HIF-1α accumulation in MDA-MB-231 cells (Figure 5E), indicating that ERK1/2 is the upstream signal that regulates HIF-1α. Moreover, U0126 treatment dramatically reduced XCL1-stimulated cell migration (Figure 5F,G). These data confirmed that activation of ERK1/2 by XCL1 participated in the HIF-1α/EMT signaling pathway, leading to an enhancement of MDA-MB-231 cell migration. Importantly, these observations were consistent with previous reports. Growing evidence has indicated that the activation of ERK signaling plays a key role in metastatic breast cancer, which may be correlated with EMT promotion [27,28,56,57]. High ERK expression levels were a negative prognostic factor in TNBC patients [58]. Moreover, ERK activation was associated with the effects of CXCL5, another chemokine, on the downregulation of E-cadherin in MDA-MB-231 and MCF-7 breast cancer cells [59]. In conclusion, our results indicated that XCL1 promoted MDA-MB-231 cell migration via activation of the ERK/HIF-1α/EMT signaling pathway.

MMPs belong to a family of zinc-dependent endopeptidases and selectively degrade various components of the extracellular matrix [36]. Additionally, MMPs regulate signaling pathways that control cancer cell survival, growth, inflammation, and angiogenesis and play critical roles in tumor invasion and metastasis [34,35]. Particularly, XCL1 enhanced trophoblast cell invasion and migration, which was correlated with increases in MMP-2 and -9 activities [60]. Additionally, these MMPs are also involved in the effect of the XCL1–XCR1 axis on cell invasion and migration of A549 cells [8] and HTR-8 trophoblast cells [60]. In this study, however, we determined that XCL1 had no significant effects on MMP-2 and -9 expressions in MDA-MB-231 cells at the studied concentrations (3–100 ng/mL) (Figure 6). Therefore, although MMP-2 and -9 participate in the signaling pathway related to cell migration and invasion induced by XCL1 in various cancer cell types, these MMPs do not appear to contribute to the XCL1-induced promotion of MDA-MB-231 cell migration.

To obtain extended knowledge of XCL1 promoting breast cancer cell migration, we investigated the effect of XCL1 on the other breast cancer cell line using SK-BR-3 cells. Interestingly, XCL1 also concentration-dependently enhanced SK-BR-3 cell migration (Figure 7A,B). Furthermore, XCL1 induced EMT with the reduced E-cadherin, and increased N-cadherin and β-catenin nuclear translocation (Figure 7C–F). Moreover, XCL1 augmented HIF-1α accumulation and ERK1/2 phosphorylation in SK-BR-3 cells (Figure 7G,H). The activation of ERK is associated with the induction of SK-BR-3 cell migration, as reported previously [61]. Again, these results indicate that activation of the ERK/HIF-1α/EMT pathway is involved in the XCL1-induced migration of SK-BR-3 cells. Since XCL1 promoted cell migration in both MDA-MB-231 and SK-BR-3 cells through activation of the ERK/HIF-1α/EMT pathway, this effect may not be specific for TNBC. Interestingly, while the levels of MMP-2 and -9 were not altered by XCL1 in MDA-MB-231 cells (Figure 6), XCL1 significantly increased the expression of MMP-2 and -9 in SK-BR-3 cells (Figure 7I,J). It is possible that the involvement of MMP-2 and -9 in XCL1-induced breast cancer cell migration may be dependent on cell type. Further studies using various breast cancer cell types with distinct characteristics may be necessary to confirm this possibility.

Next, we investigated if the activation of ERK/HIF-1α/EMT signaling was also involved in the migration of the other types of cancer cells, such as A549 and Panc1 cells treated with XCL1. XCL1 was found to increase the migration of A549 cells (data not shown), which was consistent with a previous report [8]. In contrast, 100 ng/mL XCL1 treatment of Panc1 cells for 24 h did not induce migration (data not shown). Furthermore, we found that, at the aforementioned concentration, XCL1 treatment did not significantly alter the expression of EMT markers, β-catenin nuclear translocation, HIF-1α, and phosphorylation of ERK1/2 in A549 and Panc1 cells (Figure 8). Therefore, the activation of ERK/HIF-1α/EMT signaling by XCL1 may specifically mediate the migration of breast cancer cells, including MDA-MB-231 and SK-BR-3 cells.

**Figure 9:**
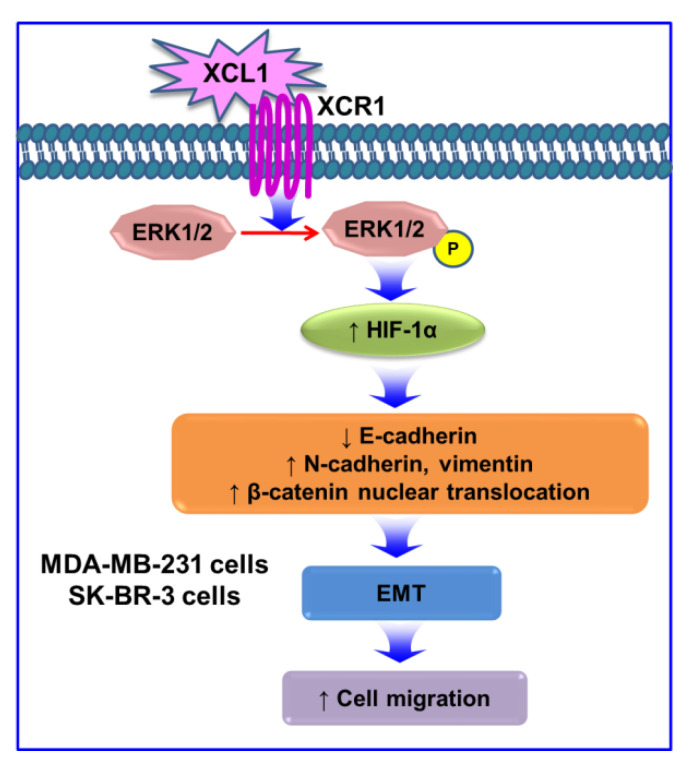
Signaling pathways involved in pro-migratory effects induced by the XCL1–XCR1 axis in MDA-MB-231 and SK-BR-3 breast cancer cells: the interaction of XCL1 with its specific receptor XCR1 enhances phosphorylation of ERK1/2, which increases HIF-1α expression. HIF-1α accumulation promotes EMT by reducing E-cadherin, by enhancing N-cadherin and vimentin, and by increasing β-catenin nuclear translocation. Activation of the ERK/HIF-1α/EMT pathway is involved in the XCL1-induced promotion of MDA-MB-231 and SK-BR-3 cell migration. XCL1, X-C motif chemokine ligand 1; XCR1, X-C motif chemokine receptor 1; ERK, extracellular signal-regulated kinase; P, phosphorylation; HIF-1α, hypoxia-inducible factor-1α; and EMT, epithelial–mesenchymal transition.

The XCL1–XCR1 axis plays multiple roles in cancer progression, depending on cancer types. XCL1 acts as a chemoattractant to recruit various immune cells such as CD4^+^ and CD8^+^ T cells, neutrophils [62], and NK cells [63]. This effect possibly augments antitumor responses, and thus, XCL1 is utilized in gene transfer immunotherapies in some types of cancer [62]. XCL1 cotransfection accentuates the therapeutic efficacy of immunotherapy using genetically modified dendritic cells with melanoma antigen gp100 [64]. Moreover, a higher serum XCL1 level at diagnosis and its progressive decreases during chemotherapy are good prognostic markers for patient survival in acute lymphoblastic leukemia [65]. In breast cancer, overexpressed XCR1 inhibits cell growth and tumorigenesis via suppression of the MAPK and PI3K/AKT/mTOR signaling pathways [10]. However, this chemokine and its receptor also promote the proliferation and cell migration of various cancer cell types such as EOC [7], NSCLC [8], and OSCC [9]. In our study, XCL1 did not induce proliferation of MDA-MB-231 and SK-BR-3 cells (data not shown). Collectively, our results demonstrate that the XCL1–XCR1 axis promotes MDA-MB-231 and SK-BR-3 breast cancer cell migration via activation of the ERK/HIF-1α/EMT signaling pathway (Figure 9). Increased expression of MMP-2 and -9 may also be involved in the XCL1-induced SK-BR-3 cell migration. To the best of our knowledge, our study is the first to demonstrate that XCL1–XCR1 interaction promotes MDA-MB-231 and SK-BR-3 cell migration through activation of the ERK/HIF-1α/EMT pathway. These results suggest that the XCL1–XCR1 axis may be a potential novel therapeutic target for the prevention of metastasis of breast cancers.

## 4. Materials and Methods 

### 4.1. Chemicals and Reagents

Fetal bovine serum (FBS), Dulbecco’s modified Eagle’s medium (DMEM), and Roswell Park Memorial Institute (RPMI-1640) medium were supplied by Corning Incorporated (Corning, NY, USA). U0126, anti-rabbit IgG and anti-mouse IgG antibodies, specific antibodies for XCR1, non-phosphorylated or phosphorylated ERK1/2, HIF-1α, E-cadherin, N-cadherin, vimentin, and MMP-2 and -9 were obtained from Cell Signaling Technology (Danvers, MA, USA). Anti-β-actin antibody was purchased from Sigma–Aldrich (St. Louis, MO, USA). Anti-lamin B1 antibody was supplied by Abcam (Cambridge, MA, USA). Alexa Fluor 488-anti-rabbit IgG secondary antibody and antibiotic–antimycotic were supplied by Thermo Scientific (Rockford, IL, USA). Recombinant human XCL1 (rhXCL1) was bought from PeproTech (Rocky Hill, New Jersey, USA). Anti-β-catenin antibody was provided by Proteintech Group, Inc. (Rosemont, IL, USA).

### 4.2. Cell Line and Culture

The human breast cancer cell lines, MDA-MB-231 and SK-BR-3 cells, and human pancreatic cancer cell line, Panc1, were supplied by American Type Culture Collection—ATCC (Manassas, VA, USA) and cultured in DMEM containing 10% FBS and 1% antibiotic–antimycotic (final concentrations of 100 U/mL penicillin and 100 μg/mL streptomycin) in an incubator at 37 °C with 5% CO_2_ until 75–80% confluency, as described in previous studies [61,66,67].

The A549 human NSCLC cell line was acquired from American Type Culture Collection—ATCC (Manassas, VA, USA) and cultured in RPMI-1640 medium containing 10% FBS and 1% antibiotic–antimycotic (final concentrations of 100 U/mL penicillin and 100 μg/mL streptomycin) in an incubator at 37 °C with 5% CO_2_ until 75–80% confluency, as published previously [68].

### 4.3. Transfection

siCtr and siXCR1 were obtained from Santa Cruz Biotechnology, Inc. (Dallas, Texas, USA). siCtr and siXCR1 were transfected into MDA-MB-231 cells using the Lipofectamine 2000 transfection reagent and Opti-MEM I reduced serum medium (Thermo Scientific, Rockford, IL, USA) according to the manufacturer’s instructions. In brief, cells were seeded in media without antibiotic–antimycotic and were allowed to reach 70–90% confluency. siRNA and Opti-MEM I were mixed to make solution A. Lipofectamine 2000 and Opti-MEM I were also mixed to make solution B. Solutions A and B were then allowed to sit undisturbed for 5 min, after which they were gently mixed to make solution C and then allowed to sit undisturbed for 20 min. Afterward, the media in cell cultures were removed and replaced with new media without antibiotics and solution C to a final siRNA concentration of 100 nM. After 6 h of transfection, the media containing siRNA were changed with new media containing antibiotic–antimycotic; media changes were performed daily. After 36–48 h, the cells were ready for further experiments.

### 4.4. Cell Migration Assays

#### 4.4.1. Wound-Healing Assay

MDA-MB-231 and SK-BR-3 cells were seeded into 24-well plates and incubated at 37 °C until they reached 75–80% confluency. These cells were treated with 3, 10, 30, and 100 ng/mL XCL1 in serum-free media for 24 h. Control cells were treated with serum-free media instead. To evaluate cell migration, cells were equally wounded with a sterile 100-μL pipette tip [69,70]. To evaluate whether XCL1 affected MDA-MB-231 cell mobility via XCR1, the cells were transfected with siCtr or siXCR1 and then treated with serum-free media (for control) or 100 ng/mL XCL1 for 24 h. The relative cell migrations over 24 h were quantified based on the changes in wound area at 0 and 24 h using a Nikon phase-contrast microscope (Nikon Instruments Inc., Melville, NY, USA) and the ImageJ software version 1.49. The cell migrations were expressed as the percentages of the respective control-treated cells.

#### 4.4.2. Transwell Migration Assay

MDA-MB-231 and SK-BR-3 cells were plated at a density of 8 × 10^4^ cells/well into 6.5-mm inserts containing an 8.0-µm pore size polycarbonate membrane from a Costar transwell system (Corning Incorporated, Corning, NY, USA). After incubating the inserts at 37 °C for 24 h, the under wells were filled with various concentrations of XCL1 (3, 10, 30, and 100 ng/mL) in serum-free media. Control cells were treated with serum-free media instead. To evaluate whether XCL1 affected MDA-MB-231 cell mobility via XCR1, the cells were transfected with siCtr or siXCR1 and then treated with serum-free media (for control) or 100 ng/mL XCL1 for 24 h. The migrated cells were fixed with 3.7% paraformaldehyde for 20 min, permeabilized with methanol for 10 min, and stained with 0.5% crystal violet for 10 min, as described in a previous study [69]. The numbers of migrated cells through the membrane were manually counted from four random fields of each sample under a Nikon phase-contrast microscope (Nikon Instruments Inc., Melville, NY, USA). Cell migrations were expressed as the percentages of the respective control-treated cells.

### 4.5. Western Blotting

MDA-MB-231, SK-BR-3, A549, and Panc1 cells were seeded into 35-mm culture dishes and incubated at 37 °C until they reached 75–80% confluency. The cells were treated with the indicated concentrations of aforementioned XCL1 for 24 h. Control groups were treated with serum-free media instead. To evaluate whether XCL1 affected the expression of E-cadherin, N-cadherin, vimentin, β-catenin nuclear translocation, phosphorylated-ERK1/2, and HIF-1α in MDA-MB-231 cell via XCR1, the cells were transfected with siCtr or siXCR1 and then treated with serum-free media or 100 ng/mL XCL1 for 24 h. The cells were then washed with cold *phosphate-*buffered** saline (PBS) and then lysed with lysis buffer on ice for 30 min. The lysis buffer (per 10 mL) contained 10 mM Tris-HCl (pH 7.5), 150 mM sodium chloride, 2 mM ethylenediaminetetraacetic acid, 4.5 mM sodium pyrophosphate, 1% (*v*/*v*) Triton X-100, 0.5% octylphenoxy poly(ethyleneoxy)ethanol (IGE-PAL CA-630), 10 mM β-glycerophosphate, 1 mM sodium orthovanadate, 1 mM sodium fluoride, and one tablet of protease inhibitor cocktail (Roche Diagnostic GmbH, Mannheim, Germany). The cell lysates were then centrifuged at 16,000 rpm and 4 °C for 30 min. The supernatants containing the extracted proteins were then collected and stored at −80° C until utilized. To evaluate the effect of XCL1 on the nuclear translocation of β-catenin, the cytosolic and nuclear extractions were separately lysed using the NE-PER^®^ Nuclear and Cytoplasmic Extraction Reagents (Thermo Fisher, Rockford, IL, USA). Electrophoresis and immunoblotting were conducted according to previously reported procedures [70,71]. Briefly, cell lysates including the equivalent amount of proteins were resolved by SDS-PAGE and then transferred to nitrocellulose membranes at 100 V for 90 min. Afterward, the transferred membranes were blocked with 5% skim milk (BD Biosciences, San Jose, CA, USA) at room temperature for 90 min, then incubated with targeted primary antibodies in 5% bovine serum albumin (MP Biomedicals, Solon, OH, USA) at 4 °C overnight. The membranes were then rinsed with Tris-buffered saline including 0.1% Tween 20 and incubated with the respective horseradish peroxidase-conjugated secondary antibodies at room temperature for 60 min. Specific bands were visualized in a Bio-Rad ChemiDoc XRS imaging system with enhanced chemiluminescent reagents (Bio-Rad, Hercules, CA, USA).

### 4.6. Immunocytochemistry

To assess the intracellular localization of β-catenin, immunocytochemical analyses were conducted following previously reported procedures [69]. Briefly, MDA-MB-231 cells were plated at a density of 1.5 × 10^4^ cells/well on coverslips in the wells of 24-well plates for 24 h. The cells were transfected with siCtr or siXCR1 and then treated with serum-free media or 100 ng/mL XCL1 for 24 h. In the next step, the cells were fixed with 3.7% paraformaldehyde for 15 min and gently washed three times with PBS. The fixed cells were then permeabilized in 1% Triton X-100 for 5 min and gently washed three times with PBS. The MDA-MB-231 cells were treated with 5% goat serum for 30 min to block nonspecific binding and incubated with anti-β-catenin antibody at a 1:200 dilution overnight at 4 °C, followed by gentle washing with PBS three times. Afterward, the cells were incubated in the dark with Alexa Fluor 488-anti-rabbit IgG secondary antibody at a 1:400 dilution at room temperature for 1 h. The coverslips were then gently rinsed with PBS and mounted with antifade mounting medium with DAPI (Vector Laboratories, Inc., Burlingame, CA, USA). Fluorescent signals were visualized using a Nikon confocal laser-scanning microscope (Nikon Instruments Inc., Melville, NY, USA).

### 4.7. Statistical Analyses

All data were displayed as the mean ± SEM from at least three independent experiments. Statistical significance was determined by one-way analysis of variance (ANOVA) using the SigmaPlot 12.5 software (Systat Software Inc., San Jose, CA, USA). *p* < 0.05 was considered statistically significant.

## 5. Conclusions

Our study determined that XCL1 dramatically induced the migration of MDA-MB-231 cells. Furthermore, XCL1 promoted EMT via the suppression of E-cadherin, the upregulation of N-cadherin and vimentin, and the nuclear translocation of β-catenin. Additionally, XCL1 enhanced ERK1/2 phosphorylation and HIF-1α expression. Notably, all these effects of XCL1 on MDA-MB-231 cell migration and intracellular signalings were inhibited by knockdown of XCR1 through siXCR1 transfection, suggesting that these activities were mediated by an XCL1–XCR1 interaction. Moreover, treating MDA-MB-231 cells with U0126, a specific inhibitor of MEK1/2, inhibited HIF-1α accumulation as well as XCL1-promoted cell migration. The levels of MMP-2 and -9 expression were not significantly altered by XCL1 in MDA-MB-231 cells. Collectively, our findings indicate that XCL1–XCR1 promotes MDA-MB-231 cell migration through activation of the ERK/HIF-1α/EMT signaling pathway. Similarly, XCL1 also induced cell migration through activation of the ERK/HIF-1α/EMT pathway in SK-BR-3 cells. The increased levels of MMP-2 and -9 may also contribute to the pro-migratory effect of XCL1 in SK-BR-3 cells. In contrast, however, activation of the ERK/HIF-1α/EMT signaling was not induced by XCL1 in the other types of cancer cells such as A549 and Panc1 cells. Based on our findings, targeting the XCL1–XCR1 axis and its intracellular signaling pathway involved in the cell migration may be a promising novel strategy to prevent breast cancer metastasis.

## Abbreviations

XCL1	X-C motif chemokine ligand 1
XCR1	X-C motif chemokine receptor 1
EOC	Epithelial ovarian carcinoma
NSCLC	Non-small cell lung carcinoma
OSCC	Oral squamous cell carcinoma
MAPKs	Mitogen-activated protein kinases
TNBC	Triple-negative breast cancers
ERK	Extracellular signal-regulated kinase
HIF	Hypoxia-inducible factor
EMT	Epithelial–mesenchymal transition
siRNA	Small interfering RNA
siCtr	Small interfering RNA targeting control
siXCR1	Small interfering RNA targeting XCR1
MMP	Matrix metalloproteinase
JAK2/STAT3	Janus tyrosine kinase 2-signal transducer and activator of transcription 3
MEK	Mitogen-activated protein kinase kinase
